# Supplementary material for: The genome sequence of the soldier beetle, Malthodes minimus (Linnaeus, 1758) (Coleoptera: Cantharidae)
Source: Wellcome Open Res. 2026 Jan 6;11:16. [Version 1] doi: 10.12688/wellcomeopenres.25397.1 (PMC12949377; doi:10.12688/wellcomeopenres.25397.1)
Supplement: Peer review history

# Reviewer report: Reviewer response for version 1

**DOI:** 10.21956/wellcomeopenres.27981.r147042

**Published:** 2026-02-27

**Authors:** Maria Antonia Madrid Restrepo

**Affiliations:** 1 KU Leuven, Leuven, Belgium

This Data Note presents a chromosome-level genome assembly for the soldier beetle *Malthodes minimus*, generated as part of the Darwin Tree of Life (DToL) project. The manuscript follows the standard DToL genome note format, providing detailed methodological documentation, quality metrics, and public data accessions. To strengthen scientific context, the authors could expand on how this species is phylogenetically informative within its clade, whether the identified chromosome numbers match expectations, if the small Y size is expected, and maybe highlight potential uses for sex chromosome evolution.

 The used protocols and methods are appropriate and represent best-practice methods for reference genome production and align with Earth BioGenome Project standards. Manual curation steps are explicitly described (119 breaks, 155 joins), which increases transparency. No methodological concerns compromise scientific validity. The assembled genome is clearly of high technical quality.

 One suggestion is for the authors to explain how they differentiate the Y chromosome from small automosomal fragments, a small sentence should suffice, explaining what methods were used for the assignment.

Are sufficient details of methods and materials provided to allow replication by others?

Yes

Is the rationale for creating the dataset(s) clearly described?

Yes

Are the datasets clearly presented in a useable and accessible format?

Yes

Are the protocols appropriate and is the work technically sound?

Yes

Reviewer Expertise:

Computational evolutionary genomics, bioinformatics, population genetics

I confirm that I have read this submission and believe that I have an appropriate level of expertise to confirm that it is of an acceptable scientific standard.

# Reviewer report: Reviewer response for version 1

**DOI:** 10.21956/wellcomeopenres.27981.r144871

**Published:** 2026-01-23

**Authors:** Hume Douglas

**Affiliations:** 1 Agriculture and Agri-Food Canada, Ottawa, Canada

The work appears to be well done. I particularly checked whether the insect identification was done well, and the authors had followed good methods. This will be useful data for phylogenies of Elateroidea and of Cantharidae, as well as other kinds of science. Such high-quality, annotated genomes are particularly useful.

Are sufficient details of methods and materials provided to allow replication by others?

Yes

Is the rationale for creating the dataset(s) clearly described?

Yes

Are the datasets clearly presented in a useable and accessible format?

Yes

Are the protocols appropriate and is the work technically sound?

Yes

Reviewer Expertise:

Beetle systematics

I confirm that I have read this submission and believe that I have an appropriate level of expertise to confirm that it is of an acceptable scientific standard.
